# Learning positive social information reduces racial bias as indexed by N400 response

**DOI:** 10.1371/journal.pone.0260540

**Published:** 2021-11-24

**Authors:** Alessandra Brusa, Antonia Pesič, Alice Mado Proverbio

**Affiliations:** Department of Psychology, Neuro-Mi, Milan Center for Neuroscience, University of Milano-Bicocca, Milan, Italy; Sapienza University of Rome, ITALY

## Abstract

The present study used EEG/ERPs to detect the activation of implicit stereotypical representations associated to other-race (OR) people and the modulation of such activation through the previous presentation of positive vs. neutral social information. Electrophysiological signals were recorded in 40 Italian Caucasian participants, unaware of the overall study’s purpose. They were presented with 285 sentences that could either violate, non-violate (e.g., “the Roma girl was involved in a robbery) or be neutral with regard to stereotypical concepts concerning other-race people (e.g. Asians, Africans, Arabic). ERPs were time-locked to the terminal words. Prior to the sentence reading task, participants were exposed to a 10 minutes colourful video documentary. While the experimental group was presented a video containing images picturing other-race characters involved in “prestigious” activities that violated stereotypical negative assumptions (e.g. a black neurosurgeon leading a surgery team), the control group viewed a neutral documentary about flora and fauna. EEG signals were then recorded during the sentence reading task to explore whether the previous exposure to the experimental video could modulate the detection of incongruence in the sentences violating stereotypes, as marked by the N400 response. A fictitious task was adopted, consisted in detecting rare animal names. Indeed, only the control group showed a greater N400 response (350–550 ms) to words incongruent with ethnic stereotypes compared to congruent and neutral ones, thus suggesting the presence of a racial bias. No N400 response was found for the experimental group, suggesting a lack of negative expectation for OR individuals. The swLORETA inverse solution, performed on the prejudice-related N400 showed that the Inferior Temporal and the Superior and Middle Frontal Gyri were the strongest N400 intra-cortical sources. Regardless of the experimental manipulation, Congruent terminal words evoked a greater P300 response (500–600 ms) compared to incongruent and neutral ones and a late frontal positivity (650–800 ms) was found to be larger to sentences involving OR than own-race characters (either congruent or incongruent with the prejudice) thus possibly indicating bias-free perceptual in-group/out-group categorization processes. The data showed how it is possible to modulate a pre-existing racial prejudice (as reflected by N400 effect) through exposure to positive media-driven information about OR people. Further follow-up studies should determine the duration in time, and across contexts, of this modulatory effect.

## Introduction

Stereotyping is a cognitive process by which a specific set of attributes and beliefs is associated to a social group (e.g., [1]). Stereotypes are embedded within cultural systems and deeply impact how people interact with each other. They are learned and reinforced by numerous processes. They develop early in life, partly through associative learning processes [2], so that even young children easily learn social stereotypes based on features that are salient for categorization such as gender, age, race or physical attractiveness [3]. If on one hand a key role is played by the influence and interactions with parents, friends and peer groups [4, 5], the impact of media in the formation and maintenance of cultural stereotypes and prejudicial feelings toward out-groups seems to be preponderant [6]. Several studies demonstrated that mainstream media has historically marginalized and underrepresented minority groups [7–10]. For instance, researchers have suggested that the negative representation of African Americans and Latinos as criminal, aggressive and unintelligent in the media help reinforce hostile prejudice against these social groups [8]. Moreover, media representations have a strong impact on identity management strategies. Saleem et al. [11] revealed that Muslim American students who viewed negative media representations of their religious group were less likely to desire acceptance by other Americans and more likely to avoid interactions with majority members. Overall, stereotypes are often embedded in the cultural system people are immersed in and are shared within a given culture [12].

Once established, stereotypes become hard to deconstruct and they can influence people’s lives in a variety of ways, shaping attitudes (prejudice) and behaviours (discrimination), influencing for instance academic performance, career choices and salary gap.

Researchers have developed several techniques to improve attitudes toward outgroups that have been found to be effective. Kawakami and colleagues [13] found that students who practiced responding in nonstereotypical ways to members of other groups became better able to avoid activating their negative stereotypes on future occasions. Moreover, when people are exposed to or are invited to think about counterstereotypic persons, for instance strong women [14] or Black role models [15], they became less prejudicial towards that social group.

Allport [1] provided one of the first contributions in understanding what are the conditions that allow stereotypes to be modulated and prejudices to be reduced in the publication "The nature of prejudice". He formulated the "Contact Hypothesis", according to which to reduce prejudices and hostilities it is best to encourage contact between members of different groups. Allport additionally identified a series of conditions to be met so that contact can produce positive effects (e.g., Equality of status, Social and institutional support, Pleasant contact and Intergroup cooperation in order to achieve a common goal).

Following this line of thoughts, if on the one hand the media can reinforce certain prejudices and stereotypes, on the other they can be used as educational tool that can raise awareness and influence attitudes towards members of different social groups to reduce intergroup prejudices [9, 10, 16].

For example, Ramasubramanian and colleagues [10] showed that activation of implicit racial stereotypes decreased when people were exposed to news stories disconfirming stereotypes and that there are media-based strategies to reduce accessibility to stereotypes. Also Soble and colleagues [17] showed that a short video could modify the racial attitudes of white college students towards peers of colour. In the experimental design students were assigned to two experimental groups: one group viewed a short documentary depicting the life of a Caucasian and an Afro American subject, followed by hidden cameras over the course of a day, the other group did not view any video. The authors found that the experimental group presented reduced prejudicial attitudes and reduced fear of minorities compared to the control group as assessed by the *Color-Blind Racial Attitudes Scale* (CoBRAS; [18]), the *Psychosocial Costs of Racism to Whites* scale (PCRW; [19]), and *the Quick Discrimination Index* (QDI; [20]).

Because stereotypes often operate out of awareness, social psychologists and neuroscientists have developed methods for assessing them indirectly. Research using implicit measures suggest that stereotypes and prejudices are easily activated when we encounter members of different social groups [21].

To overcome issues related to social desirability, researchers have effectively studied the implicit activation of stereotypes at the brain level by means of EEG/ERPs. Several studies have shown that ERPs are sensitive to violations of race- and gender-based stereotypes [22–32].

Specific attention has been paid to the N400 response, a negative deflection of the ERPs peaking around 400 ms, that indexes a difficulty in accessing and integrating incoming semantically incongruent information with previous knowledge [33]. Indeed, researchers discovered that lexical-semantic knowledge and general knowledge about the world were both integrated in mental representations in the same time interval during sentence comprehension (about 300 ms from stimulus presentation; [27]). Furthermore, the N400 response was found effective to explore the activation of conceptual/semantic associations related to world knowledge such as stereotypes using both visual or written stimuli [22–24, 27, 29–31, 34–36].

For instance, Hehman and colleagues [22] argued that the N400 component could be considered an index of stereotype accessibility. The authors used a prime-target paradigm and measured a greater N400 reactivity in response to trials in which the prime (face: Black/White) was incongruent with the stereotypical target (trait: positive/negative) than when primes and targets matched. Similarly, Wang et al. [31] found that the N400 amplitude was modulated by the congruent or incongruent association of stereotypical traits to pictures of Chinese rural workers or city dwellers. In the context of social stereotypes the N400 response might therefore index the detection of information not corresponding to available world knowledge, including social attributes stereotypically associate to a social or ethnic group.

In the realm of gender stereotypes, Proverbio and collaborators [23, 24] also used ERPs to measure, by means of a N400 response, the detection of a discrepancy between gender-based occupational stereotypes and non-stereotypical written material. According to the authors [23, 24] using complete and meaningful sentences allowed participants to access and shape more easily mental representations and to form impressions according to their own experiences. Adapting the same paradigm of Proverbio et al. [23] to the study of race-based stereotypes, Brusa and colleagues [34] also found a greater N400 negativity in response to words violating ethnic stereotypes, compared to words that confirmed them or were neutral in this regard.

Moreover, the amplitude of the N400 response was found to be sensitive to several experimental manipulations. For instance, smaller amplitudes were recorded when the incoming information was more expected and therefore easier to process [37].

Since sentence comprehension occurs accounting for one’s knowledge, context and expectations deriving from prior knowledge can influence the processing of incoming information from the earliest stages [38]. Indeed, sentence comprehension can be facilitated when the right context is provided. For instance, Hald and colleagues [39] investigated with ERPs the modulation of world knowledge comprehension by providing context. The authors found that when world knowledge incongruities (e.g. *The city Venice has many roundabouts and beautiful buildings*) were preceded by local context that made violations more acceptable (e.g., *The large and increasing amount of cyclists in the inner city of Venice had to be regulated*. *The city council decided 10 years ago to replace traffic lights with other road layouts that ease traffic flow*) the N400 was reduced compared to when the current discourse context accentuated the violation. The results fostered the claim that local discourse can modulate the integration of world knowledge information retrieved from memory.

Also Duffy and Keir [40] investigated the effect of discourse context on processing of gender stereotypes during reading. After providing adequate disambiguating context, the effect of longer reading times in response to stereotype anomalies disappeared.

The modulation of the N400 effect by providing media-based context information was studied also by Jin and colleagues [41]. The authors explored the role of the media in emphasizing racial stereotypes, as reflected by the N400 effect. In particular, the authors studied the attitude of Han Chinese towards the Uygur minority using ERPs as an index of the cognitive and neural processes associated with the activation of stereotypes. The participants, all of Han ethnicity, were subdivided into two experimental groups: a priming group viewed a video that portrayed negative aspects of the Uygur group; while the control group didn’t watch any video.

The paradigm consisted in the vision of unknown faces belonging to either the Han or Uygur ethnicity followed by a lexical decision task of positive or negative words, while recording EEG signal. The results showed that the priming group, who observed the video, unlike the control group, showed a greater N400 amplitude in response to positive adjectives associated with the presentation of Uygur faces compared to when the same faces were associated with negative adjectives.

The results demonstrated that negative media information might influence people judgments toward other groups and this effect is reflected in the deflection of the N400 amplitude.

As seen so far, previous literature has demonstrated that providing local context that is able to modulate expectation on the upcoming information and therefore facilitate the integration of semantic and social incongruities can modulate the N400 effect [38–41]. When considering social stereotypes, only few studies have investigated the role of local context on the N400 (e.g. [41]). Indeed, the study by Jin and colleagues [41] demonstrated that local context provided in the form of media-driven information is able to play an influence on attitudes towards social groups modulating the N400 response. While their study showed that negative media driven information towards a social group can increase the N400 effect when associating the same social group to positive attributes, there aren’t studies to our knowledge that have explored whether associating visual positive information to a social group could in turn reduce the N400 effect. Unlike the previously used paradigms, the emphasis here was on the possibility to measure a change in the representation of social attributes, due to the incoming of newly acquired knowledge. In other words, through the N400 paradigm we aimed at investigating whether it was possible to change mental ethnic stereotypes, by measuring their mutable effects, rather than just assessing their presence.

The paradigm used in the present study was similar to the one used by Brusa et al. [34] with sentences that could either violate (incongruent), non-violate (congruent) or be neutral with regard to stereotypical concepts concerning non-Caucasian ethnic groups. While our previous study allowed to implicitly access the neural representation of ethnic stereotypes avoiding the activation of control processes related to social desirability pressure, here the investigation aimed to confirm previous results and further explore, by means of EEG/ERPs technique, whether the previous exposure to positive and progressive social information about other-race members (e.g. Arabs, Africans, Asians, South Americans) in the form of a video documentary could modulate, in a group of Italian Caucasian participants, the access of such stereotypical representation, thus the detection of stereotypes incongruences in the subsequent sentence reading task, as marked by the N400 response.

It was hypothesized that, unlike participants in the control group who were exposed to a neutral video on flora and fauna, the participants exposed to the experimental video representing other-race individuals associated to positive attributes (e.g. a Muslim activist marching for human rights or a Black Boeing’s pilot), would present a smaller N400 effect in response to sentences that violated stereotypical assumptions compared to congruent and neutral sentences.

Based on studies on the influence of context on the N400 effect and the power of media to modulate stereotypical mental representation [9, 16, 17, 39, 41], it was hypothesized that watching a documentary could update, even if only temporarily, one’s knowledge about the social world modulating their expectations, as revealed by the N400 effect.

As previously seen in several studies (e.g. [23, 24, 26, 34]), the neural generators of the N400 response elicited by the violation of social stereotypes were hypothesized to reside in cortical structures that are involved in semantic knowledge and in brain regions connected to social cognition processes, such as the representation of social groups (e.g. ethnic groups), impression formation and processing and retrieval of information about other people (e.g. [23, 34, 42–46]). Indeed, the representation of stereotypes seems to be accompanied by the activation of some brain areas involved in mnemonic processes, in particular the temporal lobe (lateral and anterior) and the inferior frontal gyrus, and of areas belonging to the social cognition network, as the middle prefrontal cortex, the temporo/parietal junction, the superior temporal sulcus and the anterior temporal cortex [23, 24, 26, 34, 47–49]. Based on the previous literature we expected that the neural sources best explaining stereotype-dependent N400 potentials would involve regions of the social brain. The literature has especially highlighted the role of the right superior/medial frontal gyrus (SFG; BA 8) in representing social attributes and stereotypes (e.g., [42, 43]). While the typical semantic N400 response (which appears focused at centro/parietal scalp sites) seems to reflect mostly the activity of the anterior medial temporal lobe (AMTL) (e.g., [50, 51]), we hypothesized that the present anterior N400 would tap at social brain areas. Indeed N400 violations were not semantic in nature but purely based on ingroup/outgroup social categorizations. The present findings will get further new lights on the cortical dynamics of social knowledge.

Moreover, as evidenced in our previous study [34], the present study aimed to investigate post-N400 positivities related to congruence and language prediction [52, 53]. Regardless of the group assigned, a P300 effect was expected to be found in response to sentences congruent with ethnic bias compared to incongruent and neutral ones, as already found in the previous study by Brusa and colleagues [34]. The P300 response, an index of contextual updating, has been shown to reflect the integration into mental representations of semantic information that was anticipated during sentence reading [54–58] and would fit the hypothesis that biased mental representations were more easily expected in the group of participants.

Furthermore, also replicating the results of our previous study [34], a late frontal positivity was expected to be found in both groups in response to sentences involving other-race individuals compared to own-race, neutral, characters. Indeed, the late positivity is a component sensitive to the emotional value of a stimulus and is commonly found in response to emotionally relevant and arousing stimuli compared to neutral ones [59–64]. For instance, Cuthbert and colleague [59] revealed that emotionally arousing pictures evoked a larger positive amplitude compared to neutral ones, while Schacht and Sommer [64], investigating the emotional influence on visual word processing, found a late ERP effect related to emotional (positive and negative) words processing compared to neutral ones. It was hypothesized here that sentences referring to OR individuals with either a stereotypical or counter-stereotypical content would be more emotionally relevant (thus evoking a larger frontal positivity) than own-race/neutral sentences.

## Materials and methods

### Participants

Forty (22 females, 18 males) volunteers aged between 18 and 35 years (M = 22.75, SD = 2.68) and ranging in education from 12 to 18 years of study (M = 16.15, SD = 1.46), participated in the experiment. Participants were randomly assigned to but demographically balanced across two experimental groups. The data from 4 participants were discarded for excessive recording artifacts. The final EEG data analyses therefore included thirty-six (n = 36) participants, of whom 18 (age = 22.59, SD = 2) belonging to the experimental group “People” and 18 (age = 22.50, SD = 3.29) to the control group “Nature”. To avoid cultural differences, all volunteers were Italian by birth, Caucasian and with both Italian biological parents. They had normal or corrected-to-normal vision, were right handed as assessed by the Oldfield Inventory ([65]; M = 0.89, SD = 0.14) and reported no history of drug abuse or neurological or mental disorders.

Subjects were recruited through the Sona-System University portal and were granted university credits in exchange for their participation.

At the end of the experiment, participants were administered an adapted version of the Pettigrew and Meertens’ Subtle and Blatant Prejudice Scale (see [66] for the Italian translation), to assess the presence of racial bias towards immigrants. Response to the questionnaire were provided on a 5-point Likert scale ranging from ‘Not at all’ to ‘Very much’ according to the agreement with the statements presented.

All the participants expressed their understanding and written consent for the study according to the Declaration of Helsinki (BMJ 1991; 302: 1194), with approval from the University Ethics Committee (prot. n° RM-2019-178). Participants were informed of the general characteristics of the study (which was defined as an ERP study on reading). However, information about the study’s hypotheses were only provided at the end of the experiment, minimizing the social desirability bias.

### Stimuli for the video manipulation

Prior to the EEG recording involving the language task, each subject, according to the experimental group assigned, was presented with one of two video documentaries, created *ad* hoc for the study using PowerPoint.

The two documentaries were comparable in the format, differing from each other only for the image content. They were featured the presentation of 95 colourful and copyright free-images, collected from the web. Each video lasted about 10 minutes (9’42”) and was musically accompanied by a romantic piano piece (Debussy -Arabesque N° 1 -Arabesque N° 2 -Clair De Lune).

The documentary presented to the experimental group (named “People of the world”) showed images depicting the lives of multiple subjects belonging to various ethnic groups (e.g. Arabs, Afro-Americans, Roma) portrayed in situations and contexts violating discriminatory stereotypes (for instance, a female Afro-American astronaut at flight controls, a Muslim human rights activist during a protest, two Afro-American Airline pilots, an Arab woman wearing the hijab doing medical research, etc.). This type of “inverted bias” conformed to what was described in the incongruent sentences of the subsequent sentence reading task during EEG recording.

The control group viewed a documentary (named “Nature of the world”) showing images depicting beautiful natural landscapes and different animal species, excluding human beings and their modified landscapes (for instance, a fjord landscape, two rabbits in a meadow, a seaside landscape, a goat climbing on alpine rocks, desert dunes, etc.).

### Procedure for the video manipulation

This manipulation occurred 20 minutes prior EEG recording. The video-documentaries were presented in free vision on a TV monitor placed at a distance of 100 cm, while people were comfortably seated and in the absence of any EEG recording at this stage. To monitor participants’ attention while watching the documentary, they were instructed to watch the documentary attentively, because the content of the documentary would have been object of specific questions in a subsequent memory task, administered at the end of the video presentation. At this purpose, a paper and pencil questionnaire involving the mnemonic recall of some of the viewed content, based on 4 YES/NO questions (Table 1), was employed.

**Table 1:** Example of questions related to the documentary content administered to participants right after the end of the video (memory test).

#	People Documentary	Nature Documentary
1	Is there an image of a woman throwing a basketball in the documentary? (Yes)	Is there an image of two rabbits in the documentary with their noses close together? (Yes)
2	Is there an image of a policeman in the documentary stopping a car on the street and looking at the driver? (Yes)	Is there an image of a dune in the desert with a palm tree in the foreground in the documentary? (Yes)
3	Is there an image of a woman on the beach cleaning a surfboard in the documentary? (No)	Is there an image of a rattlesnake in attack position in the documentary? (No)
4	Is there an image of a musician in the documentary while playing the saxophone in a club? (No)	Is there an image of a storm that hits the beach in the documentary? (No)

### Stimuli for the language task

The linguistic task performed during EEG recording involved the reading of 285 sentences belonging to three experimental conditions based on their congruency in relation to typical ethnic stereotypes present in the Italian culture. The ethnic groups considered were various (Eastern Europeans, Arabs, Africans, Asians, South Americans, Roma) and were balanced between conditions.

The Congruent condition included 95 sentences that confirmed ethnic stereotypes, such as “Naadir is the leader of a group of REBELS” (in Italian “Naadir è a capo di un gruppo di RIBELLI”); The Incongruent condition included 95 sentences that violated stereotypes expressing notions deviating from common prejudice; e.g., ‘‘Mustafa is part of a group of ALPINISTS” (in Italian: “Mustafa fa parte di un gruppo di ALPINISTI”); The Neutral condition included 95 sentences that expressed neutral concepts relative to Italian characters; e.g., “Mirco attends a group of ACTORS” (in Italian: “Mirco frequenta un gruppo di ATTORI”).

Sentences were created in a way so that the congruency with the prejudice was made explicit only at the very end by the terminal word.

Moreover, 24 extra sentences sharing the syntactic, semantic and lexical characteristics with the previous sentences acted as target stimuli. They all ended with an animal name as the terminal word (e.g. “That man was assaulted by a TIGER”, in Italian: “Quel signore è stato assalito dalla TIGRE”) for the fictitious experimental task.

The stimuli have been previously employed in another study by the present research group [34] and contextually balanced syntactically, semantically, lexically across conditions and validated in respect to common sense prejudices for the specific population (University students in the Milan metropolitan area).

For ERP averaging of all stimulus types, EEG epochs were time-locked to the terminal words.

### Procedure for the language task

Participants were blindly and randomly assigned to either the experimental (“People”) or the control (“Nature”) group by the researchers, preserving demographical balance across groups. Regardless of the group assigned, participants underwent the identical procedure that differed only for the documentary previously presented. After positioning the EEG headset, participants were invited to seat in an electrically and acoustically shielded cubicle at 100 cm from a PC screen and were asked to fixate its centre where a dot served as fixation point.

The experimental procedure involved two distinct subsequent sessions: the viewing of the documentary followed by the mnemonic recall questionnaire (without EEG recording) and the sentence reading task (with EEG recording).

In the first session, participants were provided written instructions that invited them to attentively watch the video documentary and later to answer a questionnaire. The documentary was presented on a PC screen using *Quicktime Player* and background music was reproduced through a pair of Sennheiser (202HD model) headphones. At the end of the presentation, the brief mnemonic questionnaire was provided (Table 1) and answers were collected to assess the attentive state of participants.

In the second session, where EEG signal was recorded during the sentence reading task, participants were instructed to sit relaxed but still, avoiding any head or body movements as well as ocular saccades and blinks.

**Fig 1:**
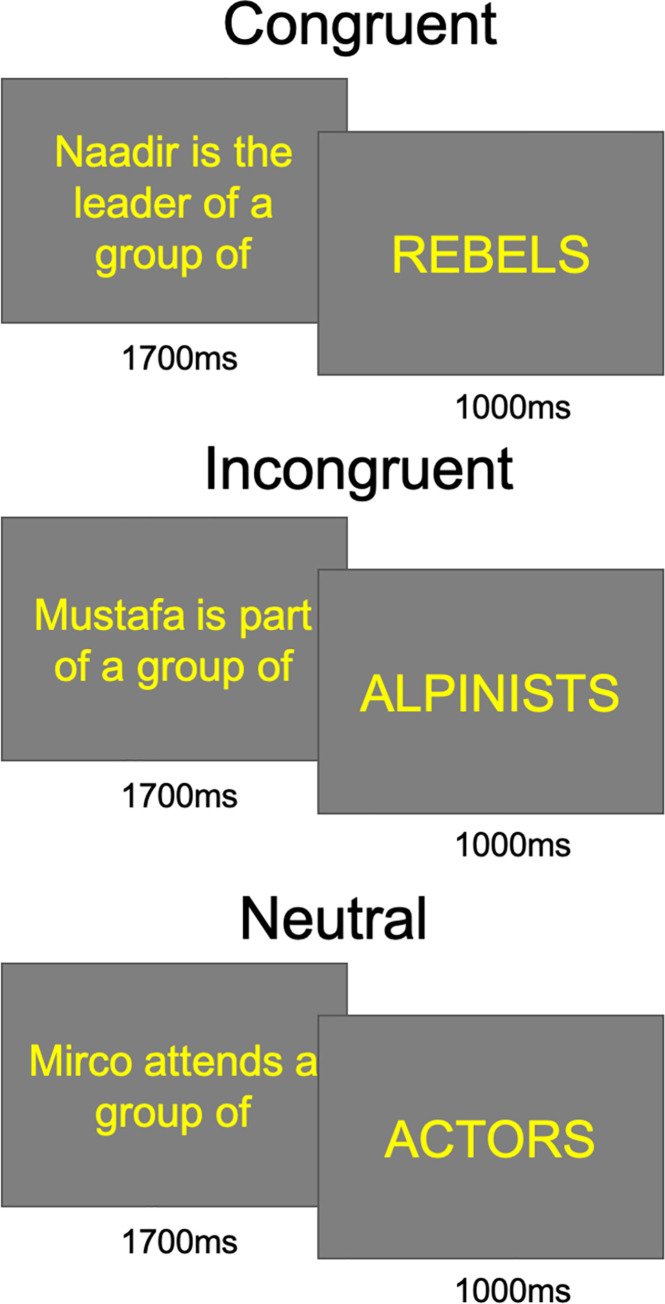
Experimental paradigm. Example of Congruent, Incongruent and Neutral sentences used in the language task. ERPs were time-locked to terminal words which were presented in uppercase for 1000 ms.

Sentences were presented with *Eevoke v2*.*2* (ANT Neuro, Hengelo, The Netherlands) in a randomized order across 8 sequences of 2 min and 40 s each interspersed with a 30-s pause. Each sentence was presented for 1000 ms at the centre of the screen arranged in a maximum of three short rows and followed, after an ISI of 700 ms, by the terminal word which was presented for 1000 ms in uppercase. The ITI was 1200 ms. The average size of terminal words was 4.47 cm x 0.8cm, implying a visual angle of 2°33’40” x 2°33’40” (minimum length = 1.5cm, maximum length = 7.9 cm). The text was printed in yellow on a grey background. An example is shown in Fig 1.

The fictitious task consisted of responding as quickly and accurately as possible to the terminal word of sentences when it contained an animal name, by pressing a joypad key with the index finger of the right or the left hand. The response hand was alternated across trials. Participants were unaware of the overall study purposes and no judgement about stereotypes was required. Response time to target terminal words was recorded only to control the attentive state and accuracy of participants.

Two training runs were administered to participants to allow them to familiarize with the procedure. A longer pause was allowed at about half the recording time. Each experimental sequence started with the presentation of 3 warning signals of 700 ms of duration (‘‘Ready, Set, Go!”) and ended with ‘‘thank you” word on the screen. Both the warnings and thanks were typed in uppercase characters.

At the end of the second session, participants were administered the Subtle and Blatant Prejudice Scale.

### EEG recording and analysis

EEG data were recorded using a standard EEG cap with 128 electrodes located according to the 10–5 International System [67] using *EEProbe v2*.*2* software (ANT Neuro, Hengelo, The Netherlands) at a sampling rate of 512 Hz (band-pass 0.016–70 Hz). Horizontal (hEOG) and vertical (vEOG) eye movements were also recorded and linked mastoids served as the reference lead. Electrode impedance was maintained below 5 KOhm. Computerized artefact rejection and manual eye-inspection was performed to remove EEG segments contaminated by ocular artefacts (saccades and blinks), muscle-related potentials, or amplifier blockages. The computerized artefact rejection criterion was a peak-to-peak amplitude exceeding 50 μV. The rejection rate was ~18% in both groups and the average number of trials per condition that went into ERP averaging was 77.58 for congruent, 78.47 for incongruent and 77.86 for neutral.

EEG epochs were synchronized with the onset of the terminal word. Evoked response potentials (ERPs) were averaged off-line from 100 ms before to 1000 ms after stimulus onset and an off-line filter (band-pass 0.16–30 Hz) was applied to the ERPs.

The mean amplitude area of the N400 response was recorded from anterior, frontal and fronto-central sites (AFz, Fz, FCz) in the 350- to 550-ms temporal window. The mean amplitude area of the P300 was recorded from anterior and fronto-central sites (AFF1, AFF2, AFz, Fz, FCz) in the 500–600-ms time window. The mean amplitude area of the frontal positivity was recorded from anterior-frontal sites (AF3, AF4, AFF1, AFF2, AFz) in the 650–800-ms time window. ERP analyses were performed using *EEprobe v2*.*2* software (ANT Neuro, Hengelo, The Netherlands). The electrode clusters and time windows were selected on the basis of previous literature [22–24, 29–31, 34, 68] and on where they matched the strongest scalp recorded activity of N400, P300 and frontal positivity potentials.

For each ERP component, the mean area amplitude values were subjected to repeated-measures ANOVAs whose factors of variability were group (“People”, “Nature”), condition (congruent, incongruent, neutral) and electrode (depending on the component of interest). Fisher post-hoc comparisons were used. The effect size for the statistically significant factors was estimated using partial etasquared (η_p_^2^) and the Greenhouse-Geisser correction was applied to account for non-sphericity of the data. All the ANOVAs were performed using Statistica software (version 10) by StatSoft. Behavioural data from the fictitious task and the mnemonic questionnaire were not statistically analysed, but response time and accuracy were collected as a record.

A standardized weighted low-resolution electromagnetic tomography (swLORETA; [69, 70]) was performed on the ERP Difference Wave relative to N400 potentials, computed by subtracting ERP response to Congruent words from ERP response to Incongruent words in the N400 time window, using ASA4 Software.

## Results

### Memory task

Behavioural data showed that the mnemonic recall of documentary contents was comparable in the two groups. On a total of four questions, the average number of correct responses was 3.61 (SD = 0.70) for the “People” group and 3.55 (SD = 0.62) for the “Nature” group, indicating a good attentive state at the documentary.

### Language task

Participants had an average Response Time (RT) to target terminal words of 625.48 ms (right hand = 627.73 ms; left hand = 625.52 ms). Moreover, the accuracy rate of responses was quite high (hits = 98%), suggesting that participants maintained an elevated attentive state throughout the experiment.

### Subtle and Blatant Prejudice Scale

The prejudice scores towards other race individuals could range from 1 (minimum prejudice) to 5 (maximum prejudice). A single overall prejudice score was computed for the two groups separately averaging all the items of the Subtle and Blatant Prejudice Scale. The mean scores were comparable in the two groups as assessed by the independent-sample t-test (t(34) = 0.40, p = 0.33) and it was 1.99 (SD = 0.35) in the “People” group and 2.04 (SD = 0.44) in the “Nature” group.

Moreover, in both groups the average score was significantly lower than the scale midpoint [3] (People: t(17) = -12.31, p < .0001; Nature: t(17) = -9.17, p < .0001), suggesting an overall low racial prejudice towards immigrants in the experimental population, regardless of the group assigned.

**Fig 2:**
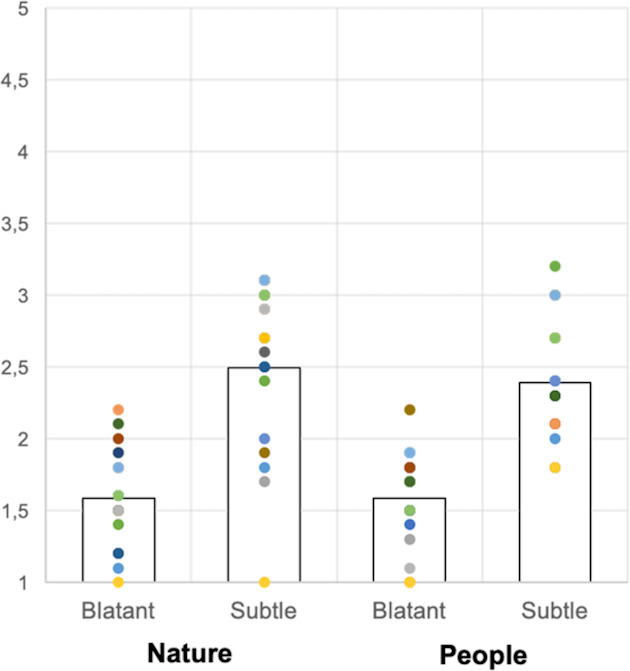
Subtle and Blatant Prejudice Scale. Scores obtained on the Subtle and Blatant Prejudice Scale by the two experimental groups. Scores could range from 1 (minimum prejudice) to 5 (maximum prejudice); for both groups, explicit prejudice (blatant) was significantly lower than the implicit one (subtle).

Considering the explicit and implicit (Blatant and Subtle) scales separately, participants in the “People” group obtained an explicit prejudice score of 1.58 (SD = 0.33) and implicit prejudice score of 2.39 (SD = 0.45), while the “Nature” group scored 1.58 (SD = 0.36) at the explicit prejudice score and 2.49 (SD = 0.59) at the implicit prejudice one. In both groups the explicit prejudice was significantly lower than the implicit prejudice (People: t(17) = -9.44, p < .001; Nature: t(17) = -9.33, p < .001). A summary of results is presented in Table 2 and Fig 2.

**Table 2:** Summary of the results of the T-test comparisons on the scores obtained at the Subtle and Blatant Prejudice Scale across the two experimental groups.

**Prejudice**	**Group**	**Descriptives**	**T Statistic**
Overall score	People	M = 1.99, SD = 0.35	t (34) = 0.40, p = .694
	Nature	M = 2.04, SD = 0.44	
Blatant Scale	People	M = 1.58, SD = 0.33	t (34) = 0.60, p = .552
	Nature	M = 1.58, SD = 0.36	
Subtle Scale	People	M = 2.39, SD = 0.45	t (34) = 0.00, p = 1.000
	Nature	M = 2.49, SD = 0.59	
**Group**	**Prejudice**	**Descriptives**	**T Statistic**
People	Blatant	M = 1.58, SD = 0.33	t (17) = -9.44, p < .001
	Subtle	M = 2.39, SD = 0.45	
Nature	Blatant	M = 1.58, SD = 0.36	t (17) = -9.33, p < .001
	Subtle	M = 2.49, SD = 0.59	

M = mean; SD = standard deviation; t = T-test.

### N400 response (350–550 ms)

**Fig 3:**
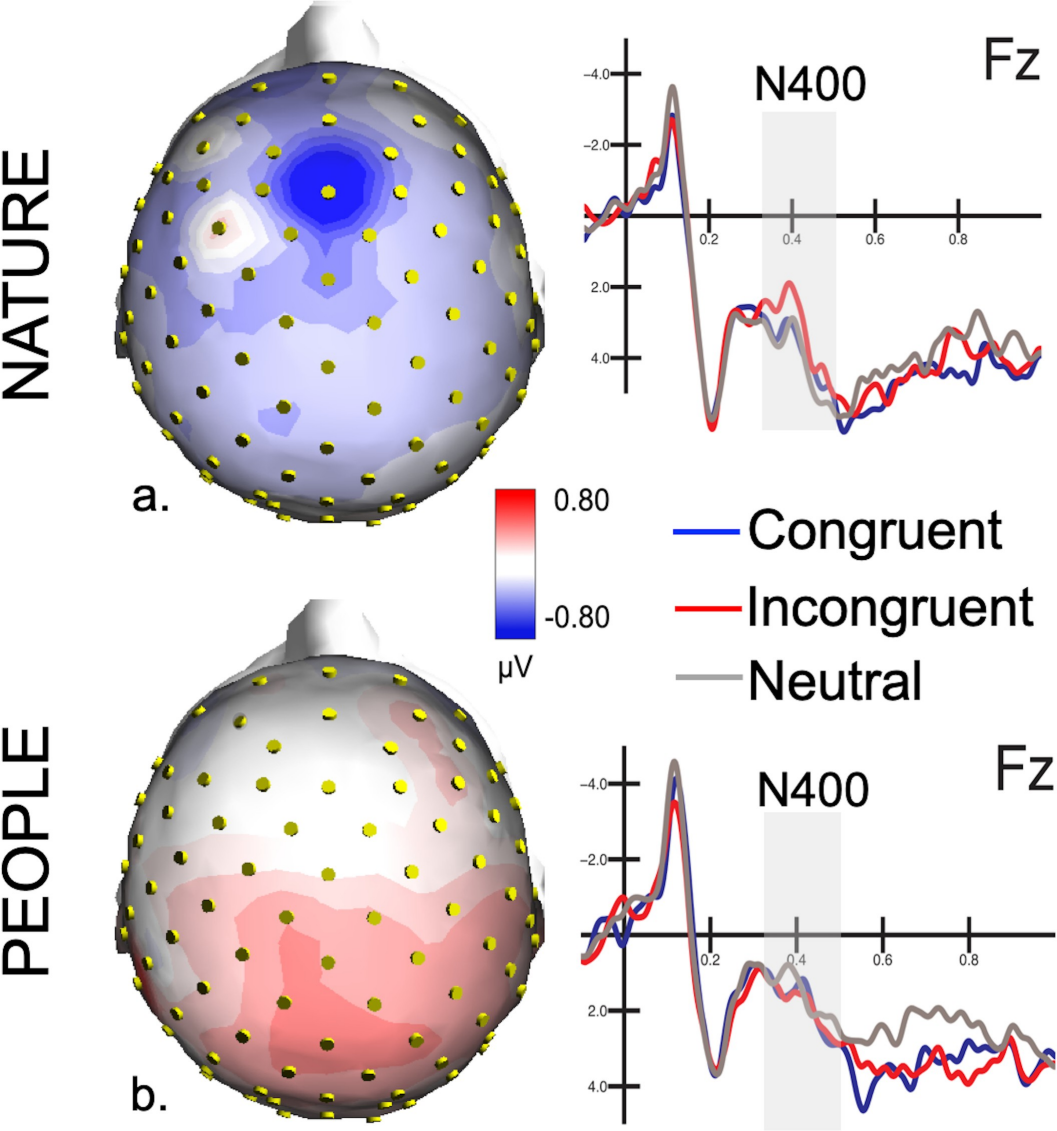
N400 topographical map. Topographical distribution of the differential voltage (Incongruent–Congruent) recorded between 350 and 550 ms post-stimulus and ERP waveforms recorded at frontal sites in response to Congruent, Incongruent and Neutral sentences; The top figure (a) shows results in the “Nature” group; the bottom figure (b) in the “People” group.

**Fig 4:**
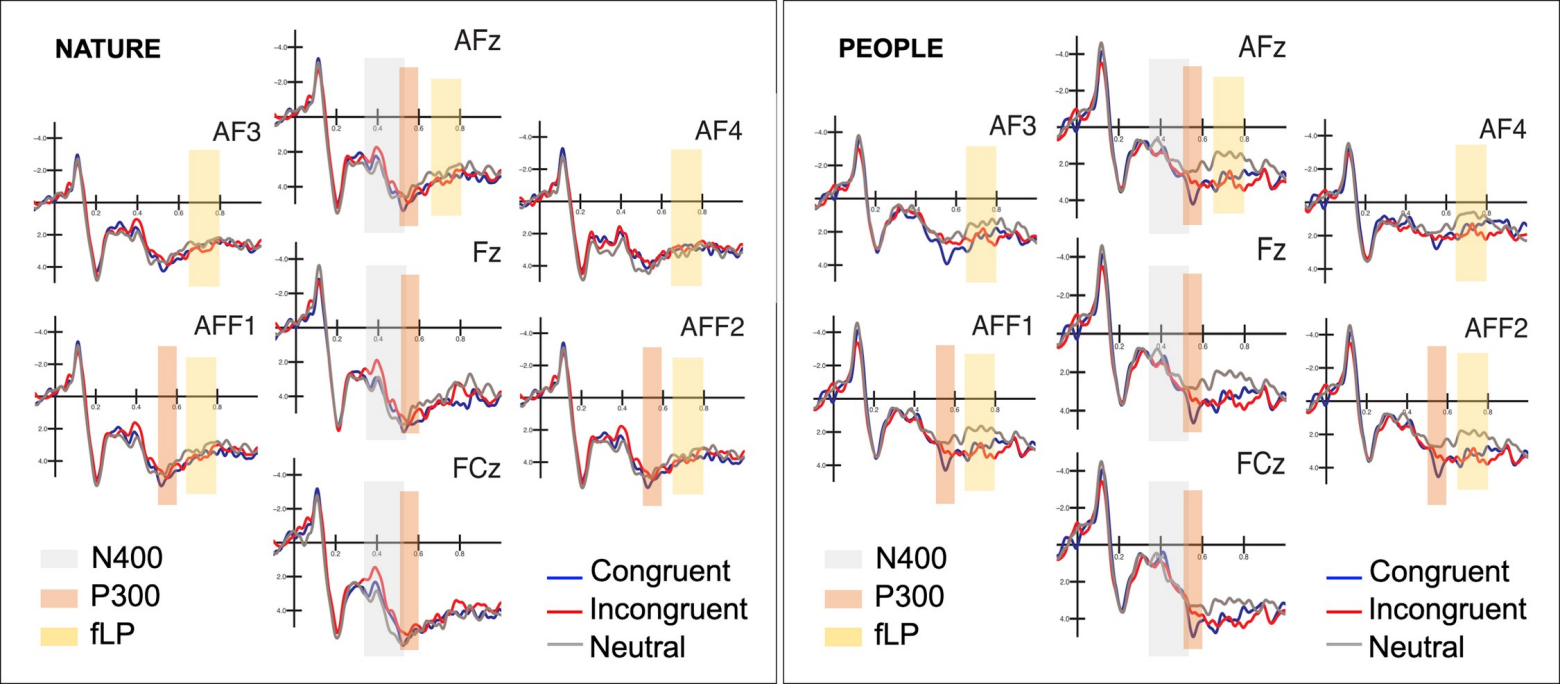
Grande average ERP waveforms recorded at anterior frontal sites in response to congruent, incongruent and neutral sentences. The time windows corresponding to the N400 (350–550 ms), P300 (500–600 ms) and frontal positivity (fLP; 650–800 ms) are highlighted. On the left recordings from the “Nature” group; on the right from the “People” group.

The ANOVA performed on the N400 amplitude values recorded over the anterior, frontal and fronto-central midline sites (AFz, Fz and FCz) in the 350–550 ms time window, revealed a significant effect of the interaction between group (“People” and “Nature”) and condition (Congruent, Incongruent, Neutral) (F(2, 68) = 3.22, p < 0.05, η_p_^2^ = 0.09). Pairwise comparisons revealed that in the “Nature” group a greater negativity (p = 0.037) was recorded in response to Incongruent (Nature: 2.11 μV, SE = 0.68; People: 1.12 μV, SE = 0.68) than Congruent (Nature: 2.85 μV, SE = 0.76; People: 0.93 μV, SE = 0.76) and also (p = 0.01) comparted to Neutral (Nature: 3.02 μV, SE = 0.78; People: 0.85 μV, SE = 0.78) terminal words, as visible in Figs 3 and 4. A similar effect has not been recorded in the “People” group (respectively p = 0.588 and p = 0.445).

### P300 response (500–600 ms)

**Fig 5:**
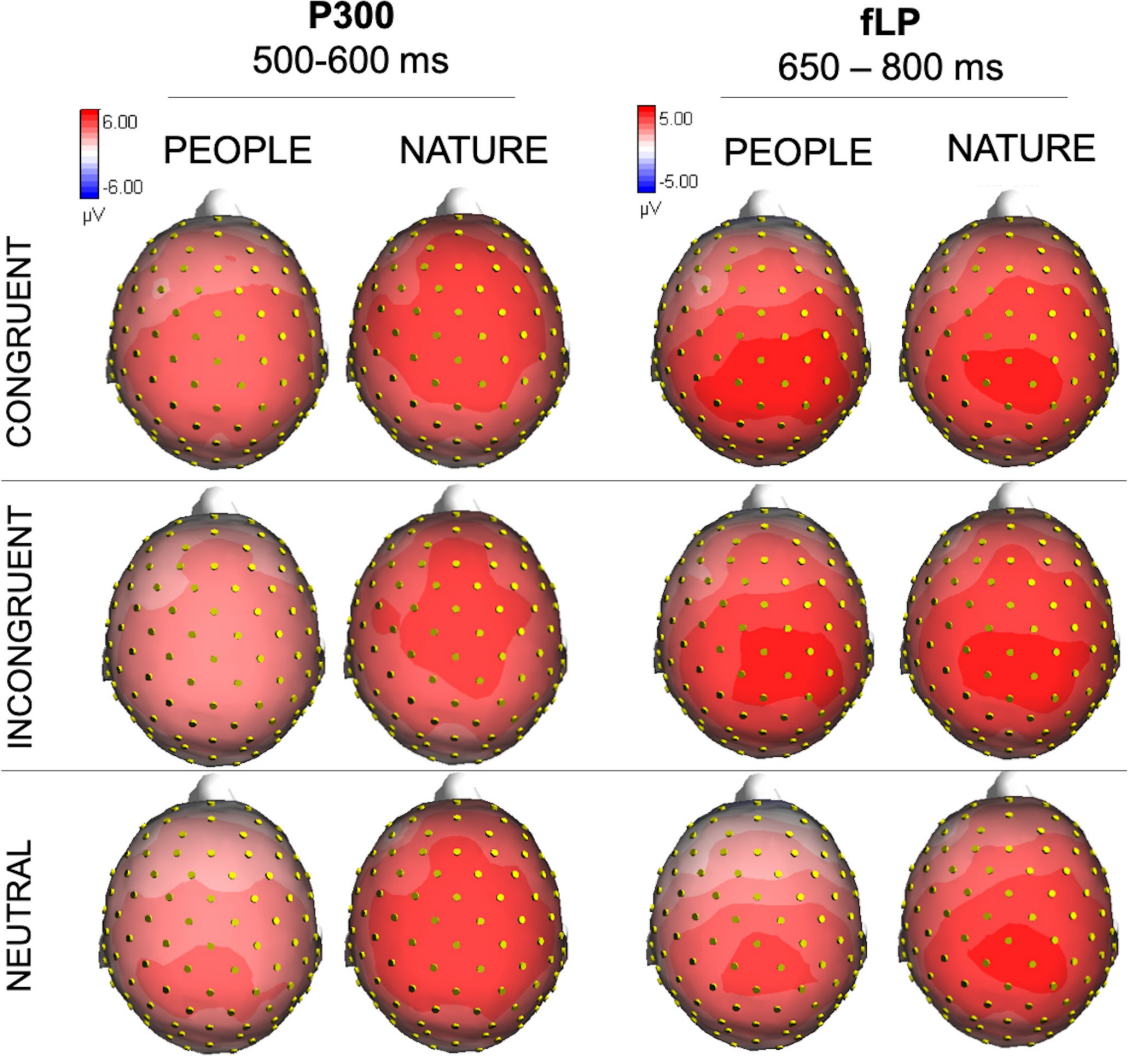
P300 and fLP topographical maps. Isocolor topographical maps of the voltage distribution (top view) of brain potentials in the time windows corresponding to the P300 (500–600 ms) and frontal late positivity (fLP; 650–800 ms).

The ANOVA performed on the P300 amplitude values, measured between 500 and 600 ms over the anterior-frontal sites (AFF1, AFF2, AFZ, FZ and FCZ) showed a borderline significant main effect of condition (F (2, 68) = 3.14, p = 0.05; η_p_^2^ = 0.08), indicating that the P300 amplitude recorded in response to Congruent terminal words (4.18 μV, SE = 0.51) was overall greater (p = 0.015) than the one measured in response to Neutral terminal words (3.41 μV, SE = 0.53). Moreover, the analysis showed a significant interaction between condition and electrode (F (8, 272) = 4.03, p <0.005; η_p_^2^ = 0.11), regardless of the experimental manipulation. Overall, the P300 amplitude was greater (p < 0.001) in response to Congruent than Incongruent and Neutral terminal words at all electrode sites; in turn the P300 response was larger (p < 0.001) to Incongruent than neutral terminal words at more anterior but not FCZ site (p = 1). P300 mean amplitude values are reported in Table 3 and showed in Figs 4 and 5.

**Table 3:** P300 mean amplitude values recorded in the 500–600 ms time window in response to congruent, incongruent, and neutral terminal words, at anterior and front-central sites (AFF1, AFF2, AFZ, FCZ, FZ).

P300 (500–600 ms)						
CONDITION	ELECTRODE	M	S.E.	C.I. -95%	C.I. 95%	N
Congruent	AFF1	4.02	0.53	2.95	5.09	36
Congruent	AFF2	4.18	0.52	3.13	5.23	36
Congruent	AFZ	3.89	0.50	2.88	4.90	36
Congruent	FCZ	4.49	0.51	3.45	5.52	36
Congruent	FZ	3.34	0.56	3.21	5.47	36
Incongruent	AFF1	3.57	0.45	2.64	4.49	36
Incongruent	AFF2	3.71	0.45	2.79	4.63	36
Incongruent	AFZ	3.44	0.43	2.56	4.32	36
Incongruent	FCZ	3.99	0.51	2.96	5.03	36
Incongruent	FZ	3.95	0.51	2.90	4.99	36
Neutral	AFF1	3.19	0.54	2.09	4.30	36
Neutral	AFF2	3.32	0.52	2.27	4.38	36
Neutral	AFZ	2.99	0.50	1.97	4.01	36
Neutral	FCZ	3.99	0.58	2.82	5.17	36
Neutral	FZ	3.53	0.57	2.37	4.69	36

M = mean; S.E. = Standard Error; C.I. = Confidence Interval; N = sample size.

Finally, the analysis revealed a significant effect of the electrode (F (4, 136) = 7.17, p <0.001, η_p_^2^ = 0.17), indicating that the P300 response was larger over the electrode FCZ (4.16 μV, SE = 0.50) compared to the electrodes AFF1 (3.59 μV, SE = 0.47; p = 0.001), AFF2 (3.74 μV, SE = 0.46; p = 0.040) and AFZ (3.44 μV, SE = 0.44; p < 0.001). The amplitude recorded over the electrode FZ (3.94 μV, SE = 0.51) was greater than the one recorded over the electrode AFZ (p = 0.008).

### Frontal positivity (650–800 ms)

The ANOVA computed on the mean area values of frontal positivity, measured between 650 and 800 ms over frontal-anterior regions (AF3, AF4, AFF1, AFF2, AFZ) didn’t reveal any difference across the two experimental groups. It showed a significant effect of condition (F (2, 68) = 4.57, p <0.05, η_p_^2^ = 0.17) such that the positivity measured in response to stimuli regarding OR individuals, either Congruent (2.61 μV, SE = 0.37; p = 0.026) or Incongruent (2.77 μV, SE = 0.36; p = 0.006), was greater than the frontal positivity recorded in response to stimuli regarding own-race individuals, that is neutral words (1.97 μV, SE = 0.40).

The analysis also revealed a significant effect of electrode (F (4, 136) = 7.30, p <0.01, η_p_^2^ = 0.18), indicating greater positivity recorded over the electrodes AFF1 (2.75 μV, SE = 0.39), AFF2 (2.91 μV, SE = 0.38) and AFZ (2.60 μV, SE = 0.36) compared to the electrodes AF3 (2.09 μV, SE = 0.37; respectively p = 0.004; p <0.001; p = 0.027) and AF4 (1.90 μV, SE = 0.35; respectively p < 0.001; p <0.001; p = 0.003). Results are showed in Figs 4 and 5.

### swLORETA source reconstruction

**Fig 6:**
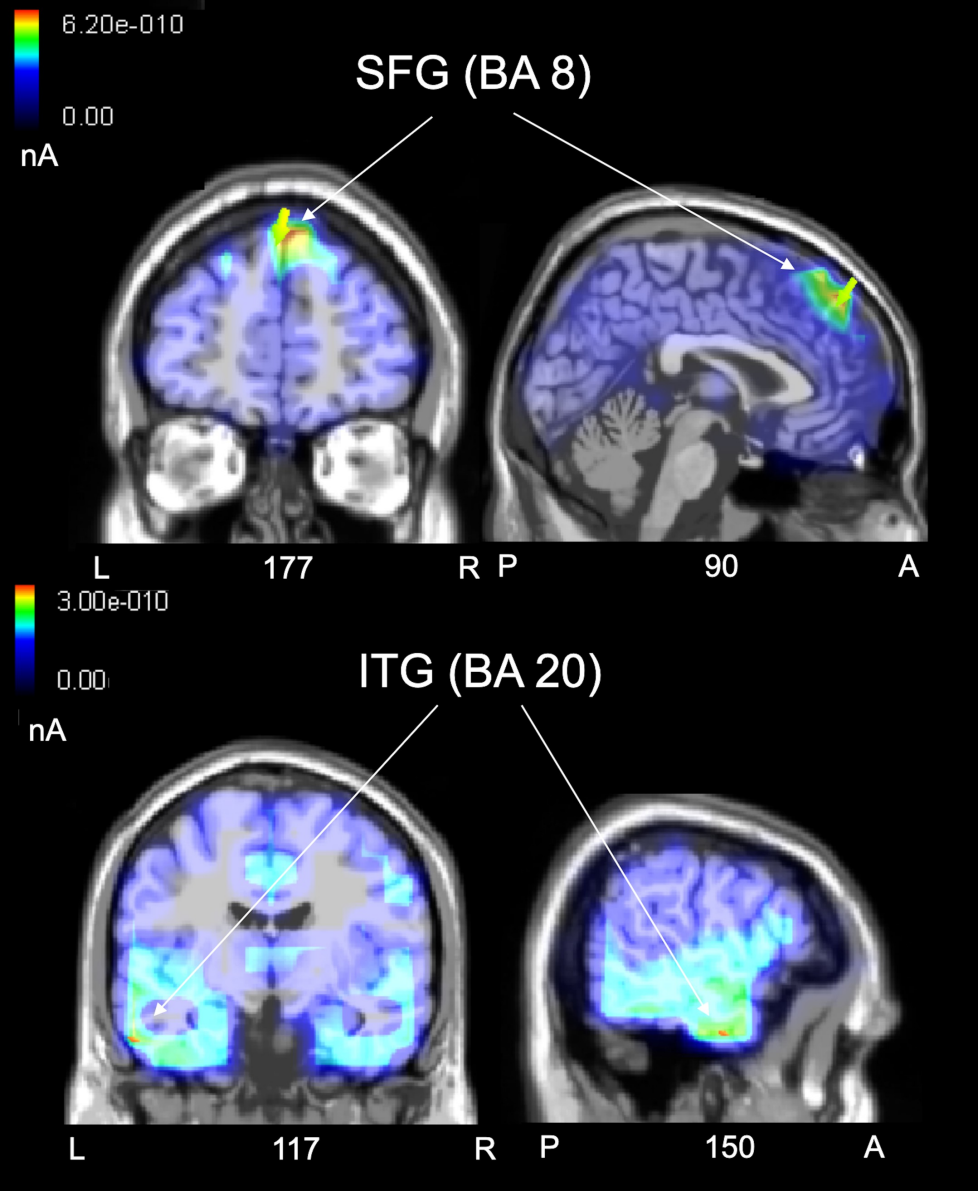
swLORETA. The most active sources relative to the ERP difference-waves (incongruent minus congruent) recorded in the 350–550 time-window (N400) in the “Nature” group were the Superior Frontal Gyrus (SFG) and the Inferior Temporal Gyrus (ITG).

The swLORETA inverse solution, applied to the Difference Waves obtained by subtracting ERP waveforms elicited by Congruent from that elicited by Incongruent stimuli in the 350–550 ms time window, corresponding to N400 component, showed (Fig 6) that the most active inner sources were the right superior frontal gyrus (SFG; BA 8) and the inferior temporal gyrus (ITG; BA 20). A complete list of all significant electromagnetic dipoles explaining the difference voltages are listed in Table 4.

**Table 4:** Source reconstruction of N400 response to stereotype violation.

Magn.	T-x	T-y	T-z	Hem.	Lobe	Gyrus	BA
6.42	1.5	40.5	50.7	R	F	**Superior Frontal**	8
2.62	60.6	-45.8	-9.5	R	T	Middle Temporal	37
2.62	-28.5	-90.3	20.8	L	O	Middle Occipital	19
2.50	-58.5	-8.7	-21.5	L	T	**Inferior Temporal**	20
2.16	40.9	-86.4	-12.4	R	O	Inferior Occipital	18

The table provides Talairach coordinates and localization of the active electromagnetic dipoles explaining the surface difference-voltage (incongruent–congruent) recorded in the 350–550 ms time window, according to swLORETA inverse solution.

Magn. = Magnitude; Hem = Hemisphere; BA = Brodmann areas; T = Talairach; R = right; L = left.

## Discussion

The present investigation aimed to explore, by means of EEG/ERPs technique, whether the exposure to a video documentary representing positive and counter-stereotypical social information about other-race members could modulate, in a group of Italian Caucasian participants, the activation of implicit stereotypical representations associated to other-race (OR) people in a language task, as indexed by the N400 response.

The pool of participants of the study, all with Caucasian background, overtly presented a low prejudice towards OR people as assessed by the Prejudice Scale. Such overall low racial bias presented differences according to the explicitness of the scale used; indeed, the implicit scale revealed slightly higher prejudice scores compared to the explicit one. This result is in line with research that posits that implicit and explicit attitudes can be dissociated [71, 72]. While explicit prejudice shapes deliberative responses which people are able to monitor, implicit attitudes are automatically activated and usually influence responses without a person’s full awareness or control [73, 74]. Therefore, these results highlight the presence, although little and below the scale midpoint, in our participants’ group of an implicit racial bias that probably bypasses explicit learnt equalitarian values.

Participants were randomly assigned to two experimental groups and accordingly presented one of two video documentaries for the experimental manipulation.

As previously hypothesized, the previous observation of the experimental video documentary was associated to a smaller N400 response, as compared to the control video. Indeed, the results showed that the “People” experimental group, which observed several representations of OR individuals associated to positive social information, presented a smaller N400 effect in response to sentences that violated common racial stereotypes compared to the “Nature” control group.

On the other hand, similarly to our previous study, showing how the N400 response signalled the existence of implicit ethnic prejudices [34], here the N400 response was larger to terminal words that were incongruent with racial stereotypes (compared to congruent and neutral ones) in the control group only witnessing a nature documentary. Therefore, the “Nature” control group, which viewed the neutral video-documentary representing landscapes and animals, showed the typical N400 effect representing the pre-existing representation of race-biased social attributes (prejudices), as it was observed in the previous study [34]. Conversely, the “People” group who observed unbiased social information (progressive documentary) presented a lower N400 amplitude.

Although a possible limit of the present study might rely in the absence of a pre-intervention measure of the N400 effect to assert that the reduced N400 amplitude in the experimental group it is truly to ascribe to the experimental manipulation, based on previous literature [23, 24, 34, 41], it is reasonable to believe that being exposed to the un-biased and counter-stereotypical video documentary played a crucial role in the reduced N400 effect.

As well-known the N400 response is an ERP component sensible to the detection of semantic and conceptual incongruities; it reflects the integration of incoming information with pre-existing knowledge and it appears larger when such integration requires an effort due to unexpected content [33]. Several studies showed that when people are faced with information (in the form of images or sentences) that are incongruent with previous conceptual/semantic associations related to world knowledge, such as counter-stereotypical social knowledge, this incongruence is reflected in a greater N400 response [22–24, 27, 29–31, 34–36]. This allowed researchers to assess the presence of implicit stereotypical mental representations in the population, revealing the sensitivity of ERPs as an implicit measure.

The present study further investigated whether these deeply grounded mental associations in the form of stereotypes could be modulated and its results are in line with the literature that highlights the role of contextual information in modulating the N400 response. Providing local context can indeed modulate expectations and facilitate the integration of incongruent content into mental representations, thus reducing the N400 effect [39–41]. Duffy and Keir [40] have shown that offering adequate context to introduce information that are incongruent with gender stereotypes modulated the N400 in response to stereotype violations, possibly facilitating their integration and reducing biased expectations. On the other hand, Jin and colleagues [41], highlighting the role of media in shaping social attitudes, proved that exposing people to videos that show negative behaviours associated to a minority group (e.g. Uyghurs) can increase biased attitudes towards the same group as showed by a greater N400 effect in response to positive trait (compared to negative trait) associations to that group. The present study explored whether media exposure could also positively shape attitudes towards ethnic minorities, attenuating social bias, using the N400 response as a neural marker of this modulation. Abundant behavioural literature confirmed on one hand the impact of media in the formation and maintenance of stereotypes and prejudicial feelings toward out-groups [6–9] and on the other hand its ability to disconfirm stereotypes and modify racial attitudes [10, 17].

According to our findings, relying on the influence of context on the N400 effect and the power of media to modulate stereotypical mental representation [9, 16, 17, 39, 41, 75], we hypothesize that watching the experimental documentary possibly updated participants’ mental representations modulating their expectations, as revealed at a neural level by the reduced N400 effect. While the effect observed was only a between-person effect and it was assessed after a short time (about 20 minutes) occurred between the video manipulation and the language task, future studies should approach the matter applying a within-person effect and eventually enquire the actual duration of such effect in the long-term memory.

Moreover, similarly to our previous ERP studies on ethnic and sexual prejudices [23, 24, 34], the swLORETA computed to reconstruct the neural generators of the N400 response elicited by the violation of ethnic stereotypes in the control group “Nature” identified a networks of cortical structures involved in semantic knowledge and social cognition processes such as the inferior temporal gyrus (ITG; BA 20) and right superior frontal gyrus (SFG; BA 8).

This pattern of results fits well with available social neuroscientific literature that posits, indeed, that stereotypical associations reside in regions that underpin semantic memory, such as the anterior and lateral temporal lobes [44, 76–78]. Moreover, in line with our results, Olson and colleagues [49] proposed that the anterior portion of the inferior temporal gyrus (the anterior temporal lobe, ATL) plays a critical role in representing and retrieving social knowledge, including biographic memory about people and more generally memory for traits and social concepts. This, according to several authors, includes stereotype representations, considered to be social semantic associations [23, 24, 44, 46, 79].

Another powerful source in terms of magnitude was the superior frontal gyrus (BA 8), a region often implicated in the representation of social attributes and theory of mind processes (TOM; [43, 80–82]). The medial prefrontal cortex (mPFC) has been found together with other regions belonging to the social cognition network to be a particularly important structure for the processing of social information (see [83, 84]). Its activity has been primarily associated with impression formation about other people and mentalizing processes [85]. Interestingly, in a recent study on the neural basis of prejudice, it was also found that the left superior frontal cortex was particularly involved in representing negative prejudices related to others [82].

Another interesting piece of data from the present study revealed, regardless of the group assigned, a greater P300 effect in response to sentences congruent with ethnic bias compared to incongruent and neutral ones, as previously seen also in the study by Brusa and colleagues [34]. The P300 response is an index of contextual updating and has been shown to reflect the integration into mental representations of semantic information that was pre-activated during sentence reading [54–58]. In line with previous literature, it appears that in the present study Congruent sentences matched participants’ previous knowledge and mental stereotypical representations, thus evoking a greater P300 response.

Similarly, a study by Correll and co-authors [29] investigated the properties of the P300 component in a racial bias experiment and demonstrated a greater P300 response in the presence of black armed subjects (compared to White armed ones) due to the automatic association of Black persons and the use of weapons. Here, both the experimental and the control group, showed a greater P300 in response to sentences that matched racial stereotypes compared to the other conditions. It is possible that, while learning through video documentary was able to facilitate the integration of upcoming stereotype-inconsistent content, hence reducing the N400 effect in response to incongruent stimuli, the P300 effect might possibly reflect that in both groups, regardless of the experimental video-manipulation, a terminal word confirmative of the stereotype was pre-activated, thus more expected, during sentence reading.

Furthermore, also in line with the results of our previous study on the measuring of implicit stereotypical mental representation [34], a late frontal positivity was found in both groups to be sensitive to the ethnicity of the characters involved in the sentences. Indeed, a greater frontal positivity was recorded in response to sentences involving other-race individuals, both in a stereotypical and counter-stereotypical way, compared to own-race neutral ones. It is possible to assume that stereotype relevant content involving OR persons might represent for the reader a more emotionally relevant and arousing material compared to neutral content involving individual of the same ethnicity. This finding fits previous literature which suggests that the late positivity is a component sensitive to the emotional and motivational value of a stimulus. It was found in response to emotional and arousing pictures, both positive and negative, [59, 60] or words [61–64] compared to neutral ones. The arousal hypothesis fits with neuroimaging literature reporting how perception of other- vs. own-race people increases the activity of amygdala, a subcortical structure that reflects arousal triggered by fast unconscious assessment of potential threat [86–88]. Moreover, in a study by Ito and colleagues [89] on social perception processes a late positive potential marked the evaluative differentiation of racial ingroup and outgroup members. Indeed, several studies have investigated the neural processes underlying ingroup-outgroup discrimination, mainly using face stimuli. For instance, Hart and colleagues [90] showed in an fMRI study that amygdala responses to human face stimuli were affected by the relationship between the perceived ethnicity of the facial stimulus and that of the observer. Moreover, greater fusiform activity has been found in response to faces of one’s own racial group [91]. ERP research examining the N170 component, an index of face encoding, revealed differential processing of ingroup versus outgroup faces, even when groups were defined arbitrarily [92]. Also, the orbitofrontal cortex has been shown to play a broader role in group-based evaluations [93].

In the present study, no significant difference was found between the two experimental groups (lack or presence of bias-free social documentary administration) in the amplitude of frontal positivity. Nonetheless, as visible in Fig 4, the effect appears stronger in the “People” experimental group. It is possible that viewing the experimental documentary depicting hundreds of individuals of OR ethnic groups might influence and increase the ingroup-outgroup discrimination. This claim isn’t supported by precise statistical data but deserves further analyses for reaching a specific conclusion. Overall, it is reasonable that adopting a larger sample size might overcome the possible limitations and strengthen the power of the present research inherent to the relatively small sample size.

### Conclusions

The present study revealed the possibility that N400 effect reflecting pre-existing racial stereotypes, might be modulated and reduced through exposure to positive media-driven information about other race (OR) people. Here, the modulatory effect on the N400 response was tested about 20–40 minutes after the experimental manipulation, so that further follow-up studies should determine its duration in time by testing it after longer periods of time, such as one week or after one month. Moreover, the present results seem to point to a sovramodal interaction in social knowledge, such that a pictorial/filmic experience could modify event related potentials in a stereotype-based language task. Hence, these data possibly suggest that the N400 response may be a sovramodal marker for conceptual incongruence (for example, [36, 94–96]) and that social knowledge is also sovramodal in nature.
